# The Effects of Nanobubble-Assisted Oxygen Supersaturation on the Growth of *Chlorella vulgaris*

**DOI:** 10.3390/mi17101169

**Published:** 2026-10-07

**Authors:** Lingxi Ouyang, Du Tuan Tran, Haotian Cha, Qin Li, Beng Hau Tan, Nam-Trung Nguyen, Hongjie An

**Affiliations:** 1 School of Environment and Science, Griffith University, 170 Kessels Road, Nathan, QLD 4111, Australia; oylx123321@gmail.com (L.O.); dutuan.tran@griffith.edu.au (D.T.T.); 2 School of Engineering and Built Environment, Griffith University, 170 Kessels Road, Nathan, QLD 4111, Australia; h.cha@griffith.edu.au (H.C.); qin.li@griffith.edu.au (Q.L.); 3 Shenzhen Institute for Advanced Study, University of Electronic Science and Technology of China, Shenzhen 518110, China; benghau@uestc.edu.cn

**Keywords:** microalgae, nanobubbles, dissolved oxygen, suppression, gas saturation

## Abstract

Algal blooms caused by excessive growth of microalgae pose serious environmental threats to aquatic ecosystems and water quality management in rivers and lakes. Oxygen saturation is important to microalgal productivity. However, effective and controllable methods to control algae growth remain a challenge in practice. In this study, we explored the effects of oxygen enrichment mediated by air and oxygen nanobubble systems on the growth of *Chlorella vulgaris*. The results revealed that oxygen enrichment treatment delayed early-stage algal growth compared to the control. With daily gas infusion, the oxygen nanobubble group with approximate dissolved oxygen concentration ~12 mg/L showed a significant reduction in final cell density (*p* < 0.01, ANOVA two-factor with replication, *n* = 3), ~2/3 of the control. Our results suggest that the reduced microalgal growth is associated with dissolved oxygen concentrations. The findings provide preliminary support for the potential applications in aquatic environments.

## 1. Introduction

Harmful algal blooms, often promoted by eutrophication, pose significant environmental threats to aquatic systems [1,2,3]. Rapidly proliferating phytoplankton can reduce water transparency, alter ecosystem functioning, and impair water quality [4,5]. Some harmful bloom-forming species can produce toxins that threaten aquatic organisms as well as animal and human health [6]. Various strategies have been developed to mitigate harmful algal blooms [7], including chemical approaches such as flocculants and algicides [8,9]. Copper sulphate is a traditional algicide for quick control of algal bloom. However, extensive utilization of copper causes copper accumulation and adverse effects on non-target aquatic life, such as fish gill damage and disruption of the aquatic food-web [10]. Some other relatively safe methods are employed to control water quality, such as biological controls involving filter-feeding organisms [11], or large aquatic plants [12], and physical or mechanical methods such as harvesting [13], continuous gas aeration [14], air flotation [15], and membrane filtration [16]. Among these, nanobubble technology has emerged as a promising solution due to its effectiveness in suppressing algal blooms with minimal secondary pollution [17,18].

Dissolved oxygen (DO) plays an important role in microalgal physiology. Previous studies have shown that elevated DO concentrations inhibit the growth and productivity of several microalgal species. High O_2_/CO_2_ ratios can alter the activity of ribulose-1,5-bisphosphate carboxylase/oxygenase (Rubisco), increasing its oxygenase activity, promoting photorespiration, and reducing net carbon fixation [19,20,21]. Oxygen supersaturation has been associated with increased respiratory energy dissipation and oxidative stress, resulting in reduced biomass in several microalgal species including *Chlorella vulgaris* [20,22]. These findings suggest that oxygen supersaturation is crucial for controlling excessive algal growth.

Nanobubbles generally refer to gas-filled voids in solution with diameters around 100 nm [23]. Due to their extremely small size, the buoyant force acting on them is counteracted by Brownian motion, allowing them to remain suspended in aqueous environments with remarkable stability [24]. Nanobubbles have a large specific surface area and a strong zeta potential [25,26]. These unique properties allow them to be used widely in many practical applications, from drug delivery [27,28,29] and wastewater treatment [30] to agriculture and aquaculture [18,31]. Nanobubbles have the potential to generate and maintain oxygen-enriched conditions in aquatic environments, thereby influencing algal growth dynamics.

Recent research studies have demonstrated the promising potential of ozone nanobubbles for controlling harmful algal blooms [17]. However, practical implications using ozone nanobubbles face some challenges [32]. First, the preparation of ozone nanobubbles raises a risk to workers who might be subjected to inhalation of ozone. Second, precise control of ozone dosage is non-trivial, as overdosing could unintentionally harm non-target organisms such as fish and aquatic plants or aerobic bacteria that inhibit algae [33]. Third, ozone may react with bromide ions in natural waters to produce bromate, a potentially carcinogenic byproduct [34]. These limitations motivate the investigation of alternative nanobubble systems. Oxygen nanobubbles are small, relatively safe and stable in water, and they may facilitate sustained oxygen transfer compared with conventional gas delivery approaches. Therefore, we hypothesize that oxygen nanobubble treatment could establish oxygen-enriched conditions and suppress algal growth at the early stages of bloom formation. However, the influence of oxygen-enriched nanobubble treatments on microalgal growth remains poorly understood.

In this study, we selected *Chlorella vulgaris* as the model microalgae to investigate the effects of oxygen-enriched conditions generated by nanobubble treatment on algal growth. Nanobubble-enriched culture solutions were prepared using a nanobubble generator, with three experimental groups: air nanobubble solutions, oxygen nanobubble solutions, and a control group without nanobubbles. During the experiment period, in one experiment group, we ran a specific gas through a nanopore nozzle to maintain a certain level of DO. We evaluated the cell accumulation of *Chlorella vulgaris* across these groups over time. In addition, the temporal variations in DO and dissolved CO_2_ concentrations were analyzed to explore their influence on the balance between photosynthesis and respiration.

## 2. Materials and Methods

The *Chlorella vulgaris* microalgal strain (CCAP 211/11S, codename CS-41) was obtained from the Commonwealth Scientific and Industrial Research Organisation (CSIRO, Clayton, VIC, Australia). BG-11 culture medium was obtained from the Australian Rivers Institute (ARI) at Griffith University (Nathan, QLD, Australia).

### 2.1. Nanobubble Generation and the Preparation of the Culturing Solution

Air and oxygen nanobubbles were generated using a nanobubble generator (Wuxi Zhengben Qingyuan, Wuxi, China). A nanobubble generator generates nanobubbles based on jet aeration through the Venturi effect. As liquid passes through a constricted section, a local pressure drop draws gas into the circulating liquid stream. The gas–liquid mixture is subsequently subjected to intense shear and pressure changes, leading to bubble fragmentation and the formation of nanobubbles. Although nanobubble generation involved cavitation, the generator has a built-in cooling system to dissipate heat generated during operation. The actual temperature of the resulting solution was observed ~15 °C, cooler than room temperature after operation, and therefore temperature effects were considered negligible. Before use, the inlet and outlet of the generator were immersed in 1 L of deionized water and operated for 10 min to rinse the entire system. This cleaning procedure was repeated three times to ensure the system was thoroughly cleaned and contaminant-free. To prepare the air nanobubble culturing solution, the BG-11 stock solution was diluted with Milli-Q water at a ratio of 1:49 to obtain 1 L of culturing medium. The prepared medium was fed into the nanobubble generator and operated for 10 min, with the gas inlet left open to the ambient environment to allow the formation of air nanobubbles. Similarly, pure oxygen gas from an oxygen concentrator (DEDAKJ DE-1S, Shenzhen Deda Health Co., Ltd., Shenzhen, China) was fed to the nanobubble generator at a flow rate of 1 L/min to generate oxygen nanobubbles in 1 L of the prepared BG-11 medium. Untreated BG-11 medium was used as a control. It should be noted that trace impurities, e.g., non-gaseous particles, originating from the culture medium or gas source cannot be completely excluded. Such impurities can present in characterizations and influence nanobubble stability.

### 2.2. Cultivation of Chlorella vulgaris Under Different Nanobubble Conditions

**Figure 1:**
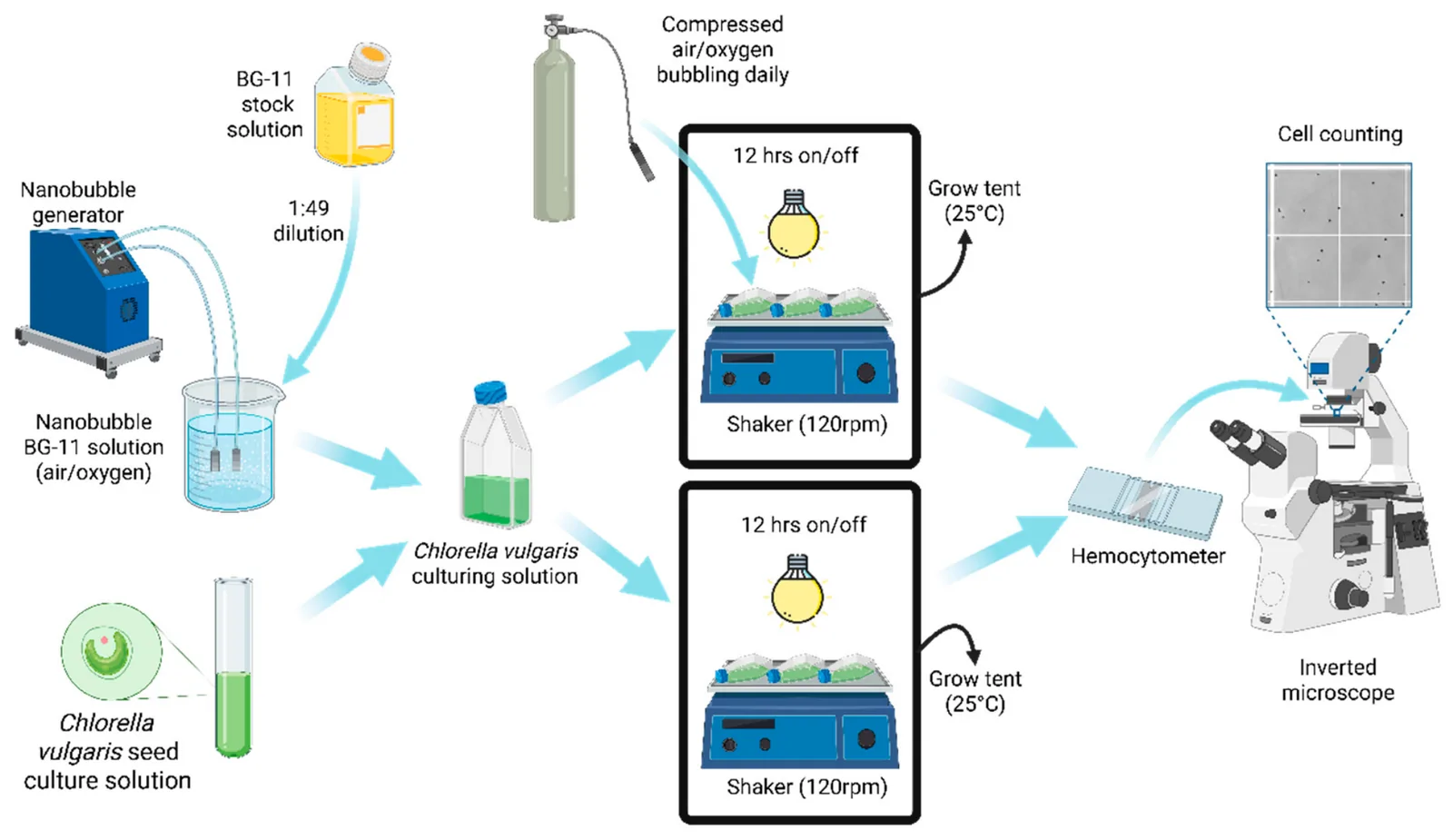
Schematic of preparation of nanobubble culturing solution, cultivation of *Chlorella vulgaris* and cell counting procedure (Made by BioRender).

The cultivation of *Chlorella vulgaris* was conducted in two experimental groups, as shown in Figure 1. Each group consisted of triplicate samples of air nanobubble culturing solutions, oxygen nanobubble culturing solutions, and a control culturing solution without nanobubbles. In the first group, the culture medium was prepared using the respective nanobubble or control solution only on Day 0. In the second group, in addition to initial medium preparation with the respective solutions, gas infusing was performed once a day to maintain DO at a certain level in the culture media. For each culturing, 30 mL of *Chlorella vulgaris* seed culture was diluted at a ratio of 1:10 with the respective nanobubble or control culturing solution and mixed thoroughly in sterile culture flasks. The initial cell concentration was adjusted to approximately 1.2 × 10^6^ cells/mL. All sample flasks were placed horizontally on an orbital shaker (NSP-300, Hanhua Intelligent Technology, Taizhou, China) at an rpm of 120 and subjected to illumination from LED grow lights, with a daily cycle of 12 h of light followed by 12 h of darkness. All sample culturing took place in an enclosure to prevent any external environmental effects. For the gas infusion group, the gases (air or pure oxygen) were infused into the culture media for 5 min (shaker was set off) once per day using sterile nanoporous diffusers (220 nm porous titanium). This approach aimed to sustain the desired dissolved gas levels throughout the cultivation period. In contrast, the other group received no additional gas input during the cultivation period.

### 2.3. Characterization of Nanobubble Solutions

The size distribution of the generated nanobubbles in the culturing solutions was characterized by a nanoparticle tracking analysis (NTA) system (Nanosight NS300, Malvern Panalytical, Malvern, UK) and a dynamic light scattering (DLS) instrument (LiteSizer 500, Anton Paar, Graz, Austria). DO and dissolved CO_2_ were measured daily, immediately post-infusion for the gas infusion group. Triplicates were used for each condition, and averages were presented with standard error of the mean. The DO level was measured with a commercial oxygen meter (Smart Sensor AR8406, Graigar Technology, Shenzhen, China). The oxygen meter probe was calibrated using sodium sulphite solutions for a zero-point oxygen concentration, and exposed to ambient air for the 100% saturation point before use. Dissolved CO_2_ was measured with a commercial dissolved CO_2_ meter (Nate8732, Nate instruments, Guangzhou, China) and the probe was calibrated before use.

### 2.4. Cell Counting of the Chlorella vulgaris

To prepare the samples for cell counting, 20 µL of culture solution was first diluted with 180 µL of Milli-Q water in a 1 mL Eppendorf tube. The mixture was thoroughly vortexed for 1 min to ensure uniform dilution. Subsequently, 10 µL of the diluted solution was carefully loaded between a glass coverslip (22 mm × 30 mm × 0.4 mm) and a Neubauer hemocytometer. The hemocytometer was then placed under a Nikon Ti2 inverted microscope (Nikon Corporation, Tokyo, Japan). Cell images were captured using an Eclipse digital camera (Nikon Corporation, Tokyo, Japan) under a 10× objective lens. ImageJ software (v1.54p) [35] was used to analyze the acquired images, enabling accurate cell counting and final determination of cell concentrations in the culture samples.

Cell density (cells/mL) was calculated based on the average cell count using the following equation:

$$P=\frac{D\times N}{V_{C}}$$

where *P* represents the cell density (cells/mL), *N* is the average number of counted cells, and *D* is the dilution factor. For this case, the dilution factor is fixed at *D* = 10. *Vc* is the volume of the counting chamber on the hemocytometer, defined by an area of 1 mm^2^ and a depth of 0.1 mm, resulting in *Vc* = 0.0001 mL.

### 2.5. Statistical Analysis

Statistical analysis was performed using ANOVA two-factor with replication in Excel. The triplicate results of cell density, DO and dissolved CO_2_ were analyzed at the level of 0.05. For repeated measures using the same flask for consecutive observation days, a linear mixed-effects model was used, in which treatment and day were included as fixed effects and flask was included as a random effect (Cell density ~ Treatment × Day + (1|Flask)). Effective size was calculated using the equation $partial\;\eta^{2}={SS}_{treatment}/\left({SS}_{treatment}+{SS}_{within}\right)$.

## 3. Results

### 3.1. Characterization of Nanobubbles

**Figure 2:**
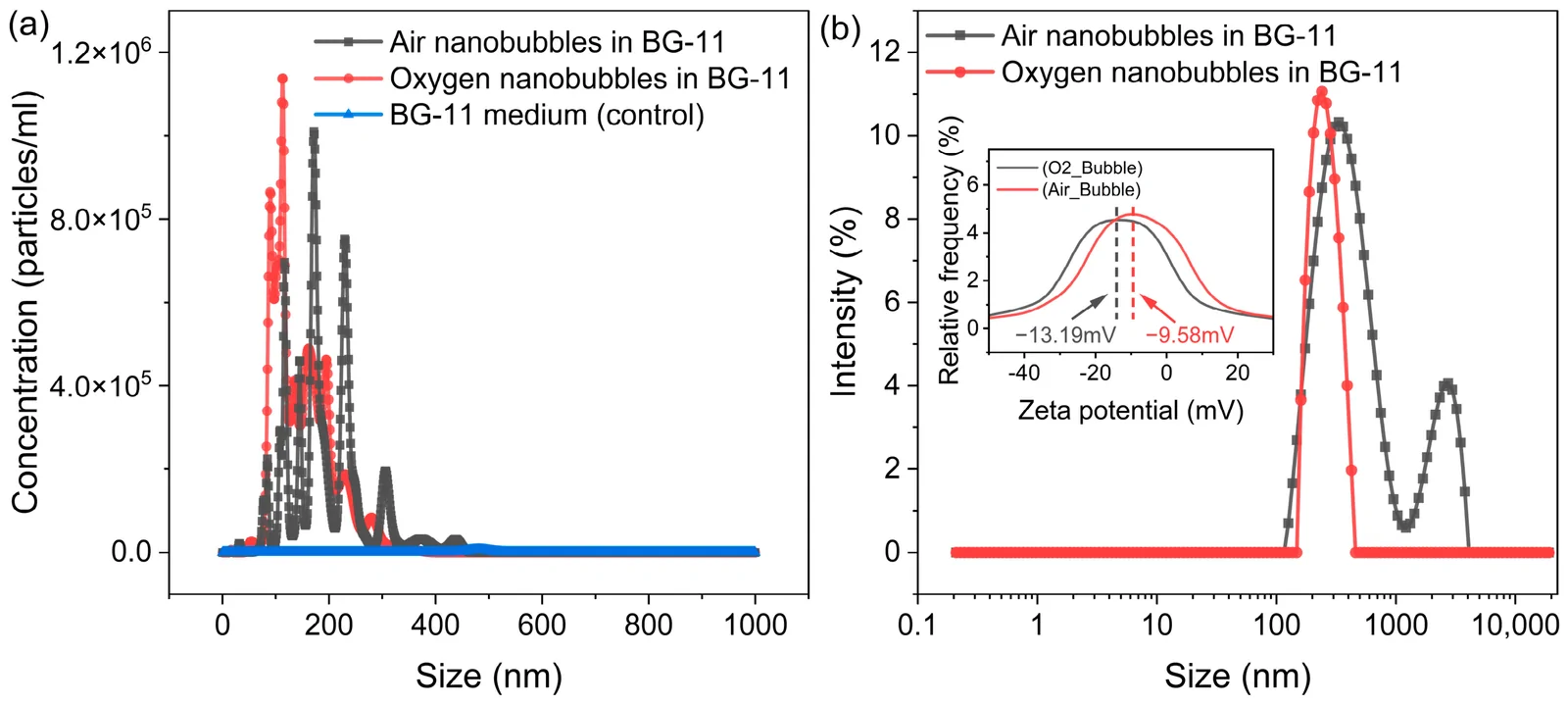
(**a**) The nanoparticle tracking analysis (NTA) size distribution and (**b**) the dynamic light scattering (DLS) results for the oxygen nanobubble solution and air nanobubble solution. The inset is the zeta potential of the oxygen nanobubble solution and the air nanobubble solution.

Before the cultivation of *Chlorella vulgaris*, the fresh culturing solutions containing nanobubbles were characterized to determine the concentration of nanobubbles present in the media. NTA results (Figure 2a) showed that both air and O_2_ nanobubble culturing solutions exhibited a particle size distribution around 150 nm; the mean size of the air nanobubbles was 202.7 ± 10.4 nm, and the mean size of the O_2_ nanobubbles was 146.3 ± 8.9 nm. Meanwhile the air nanobubble culturing solutions exhibited wider distribution and possessed three peaks at around 160 nm, 190 nm, and 220 nm. The NTA measurements suggest a similar total concentration for the air nanobubbles and oxygen nanobubbles, with a value of 6.64 × 10^7^ ± 1.18 × 10^7^ particles/mL for the oxygen culturing nanobubble solution and 6.32 × 10^7^ ± 8.0 × 10^6^ particles/mL for the air nanobubble culturing solution. A larger size distribution was observed in the air nanobubble culturing solution.

Since NTA can only measure particles up to 1 µm, we used DLS to characterize the size distribution of nanobubbles in the culture medium, covering a size range up to 10 µm. Figure 2b presents the intensity distribution of nanobubbles of varying sizes in the air and oxygen nanobubble culturing solutions. Oxygen nanobubbles in culture medium are similar to the NTA results. However, the air nanobubble solution exhibited a bimodal distribution with a broader intensity peak in the submicron range and a secondary peak around 2–3 µm. The polydispersity index (PDI) of the air nanobubbles in the BG-11 sample is around 0.24. This suggests that ambient air easily forms larger bubbles. In contrast, the oxygen nanobubble solution predominantly contained bubbles in the smaller size range of 100–300 nm, with a PDI of 0.2, indicating better size uniformity and stability. The inset shows that both types of nanobubbles have negative zeta potentials. The zeta potentials of the oxygen nanobubble solution are −13.19 mV, and the air nanobubble solution is −9.58 mV. The zeta potential shows those nanobubbles are negatively charged.

The air nanobubble treatment showed a broader size distribution and an additional population of larger particles in the micrometer range. One possible explanation is the presence of airborne particulate matter in ambient air, which may influence bubble nucleation, coalescence, or the measured particle size distribution. In contrast, the oxygen supplied from the oxygen concentrator was passed through the concentrator system and subsequently through water at the outlet, which may have reduced particulate contaminants. However, particle concentrations in the gas streams were not measured in the present study, and therefore the mechanism responsible for the observed difference in bubble size distributions remains uncertain.

In comparison, the BG-11 culture medium used for the control group was also characterized using NTA. As shown by the blue triangles in Figure 2a, no noticeable peak was observed in the size distribution profile, indicating the absence of detectable nanoscale particles in the BG-11 medium. These results support the use of BG-11 medium as a valid control for subsequent experiments.

### 3.2. Algal Growth

**Figure 3:**
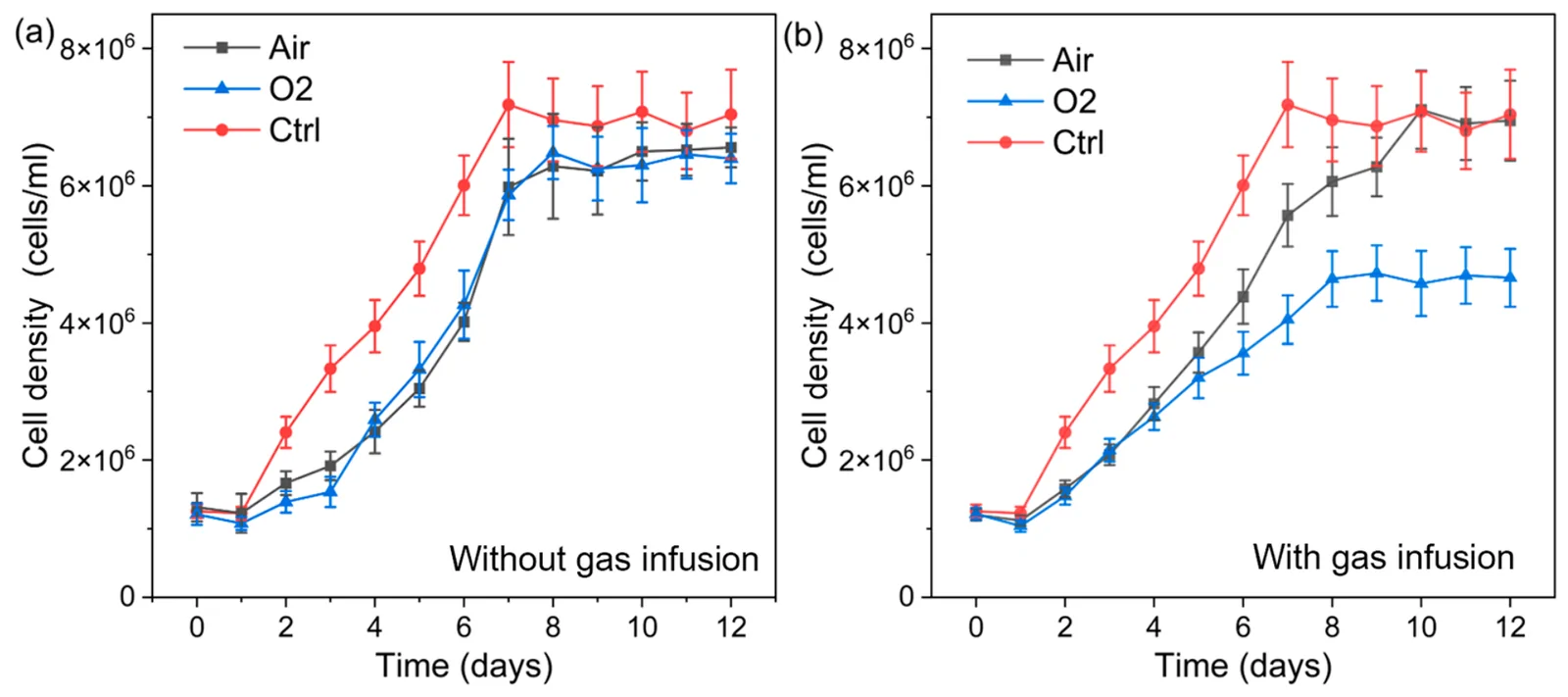
The growth profile of *Chlorella vulgaris*: (**a**) the cell number profile of air nanobubble, oxygen nanobubble, and free control culture solution without gas infusing; (**b**) the cell number profile of air nanobubble, oxygen nanobubble, and free control culture solution with daily gas infusion. The data are included in Supplementary Materials Tables S9 and S10.

Our initial hypothesis proposed that nanobubbles, particularly oxygen nanobubbles, could create an oxygen-rich environment in the culture medium and thereby inhibit the growth of Chlorella vulgaris. To test this hypothesis, we first conducted cultivation experiments mixed with nanobubbles once. Three types of culture media were used: air nanobubble-treated mediums, oxygen nanobubble-treated mediums, and a control medium without nanobubbles. All the sample groups were placed on an orbital shaker at 120 rpm during the growth period to prevent coagulation and non-uniform growth, since static cultivation will slow down the growth [36]. As shown in Figure 3a, both nanobubble-treated groups slowed down the growth rates and reduced cell density when compared to the control over the entire cultivation period. By Day 8, the cell concentrations in both the air and oxygen nanobubble groups reached a plateau at approximately 6 × 10^6^ cells/mL, which was lower than that of the control group (7 × 10^6^ cells/mL). Notably, during the early phase of cultivation (from Day 0 to Day 3), the oxygen nanobubble group showed a lower cell density than the air nanobubble group. However, from Day 4 onward, the cell concentrations of the two nanobubble groups became comparable.

These findings suggest that both air and oxygen nanobubbles exert inhibitory effects on the growth of *Chlorella vulgaris* during cultivation. The slightly lower cell density observed in the oxygen nanobubble group during the first four days suggests a retardation due to the higher initial DO level in the medium. After approximately four days, the depletion of excess dissolved oxygen likely allowed the system to reach a new equilibrium, enabling the cultures to exit the lag phase and enter exponential growth. In the late phase of cultivation (>Day 8), both nanobubble-treated groups reached a stationary phase, and cell densities no longer increased. The final concentrations appeared slightly lower than those in the control group, the difference was within the expected experimental error range during late-stage measurements. The statistical analysis showed significance between nanobubble groups and the control group (ANOVA two-factor with replication, *n* = 3; *p* < 0.01), indicating that the nanobubble treatments had a statistically significant effect compared with the control. These results indicate that nanobubbles caused a temporary delay in *Chlorella vulgaris* growth but were insufficient to impose sustained inhibition. Based on these observations, we designed a follow-up experiment incorporating daily gas infusion to maintain nanobubble concentrations.

The detailed comparative analysis enclosed in Tables S1–S8 in the Supplementary Materials contain the complete ANOVA statistics, including F values, degrees of freedom, effective size, exact *p*-values, and treatment × time interactions. In the absence of gas infusion (Table S1), Air versus Control showed significant treatment (F_1,52_ = 48.06, *p* = 6.34 × 10^−9^), time (F_12,52_ = 87.59, *p* = 4.34 × 10^−30^), and treatment × time interaction effects (F_12,52_ = 2.67, *p* = 0.0071), and partial η^2^ = 0.48. As shown in Table S2, O_2_ versus Control also showed significant treatment (F_1,52_ = 51.97, *p* = 2.30 × 10^−9^), time (F_12,52_ = 90.08, *p* = 2.2 × 10^−30^), interaction effects (F_12,52_ = 2.11, *p* = 0.032), and partial η^2^ = 0.5. In contrast, Air versus O_2_ treatment (Table S3) were observed no significant between treatments (F_1,52_ = 1.42, *p* = 0.24), while time (F_12,52_ = 1856.30, *p* = 5.91 × 10^−64^) and treatment × time interaction effects (F_12,52_ = 3.70, *p* = 0.00047) were significant.

It would be ideal to retain nanobubble concentration in cell culture during the cultivation time. However, due to the specialized design of the nanobubble generator, it was not suitable to continuously generate nanobubbles by directly flowing the growing microalgal cultures through the nanobubble generator, which could introduce adverse effects on microalgae and risks for comparison among groups. The replacement of medium by fresh nanobubble-treated medium during collection of the microalgae raised concerns of disturbance and damaging the microalgae by the filtration or centrifuge process. Instead, we considered maintaining a certain level of dissolved oxygen by daily infusing the air/oxygen into the cell culture media. Thus, gas infusion was employed once a day to maintain the level of dissolved oxygen in the culture medium. Nanoporous diffusers were used to introduce the corresponding gases (air or pure oxygen) into the nanobubble-treated media. This process is also similar to a real-world industrial lake cleaning process using nanobubbles by dipping a nanobubble nozzle in water.

Figure 3b presents the daily cell count data of *Chlorella vulgaris* with gas infusion. The results show that the control group entered exponential growth after a one-day lag phase and reached a stationary phase around Day 7, with cell density stabilizing at approximately 7 × 10^6^ cells/mL. In contrast, both nanobubble-treated groups with daily gas infusion exhibited a clear retardation in cell density. The air nanobubble group showed a delayed onset of algal growth during the initial days of cultivation, followed by gradual adaptation to the culture environment, and ultimately reached a stationary phase by Day 10, with a final cell density level comparable to that of the control. The oxygen nanobubble group, however, demonstrated a more pronounced inhibition effect. Microalgal cultures in this group reached the stationary phase around Day 8, but the final cell density was only about two-thirds (4.5 × 10^6^ cells/mL) of that observed in the control and air nanobubble groups. Both air and oxygen nanobubble groups with gas infusion are significantly different at the 0.01 level from the control group with a *p*-value < 0.01 (ANOVA two-factor with replication, *n* = 3). Under gas infusion conditions, the Air versus O_2_ treatment (Table S6) (F_1,52_ = 274.03, *p* = 2.24 × 10^−22^), time (F_12,52_ = 291.68, *p* = 3.18 × 10^−43^), and treatment × time interaction effects (F_12,52_ = 19.10, *p* = 5.58 × 10^−15^) were observed to be significant. The effective size partial η^2^ = 0.84, suggesting approximately 84% of the variance not attributable to random error was associated with the treatment effect. These observations suggest that exposure to oxygen nanobubbles in a supersaturated level of DO does not only delay the onset of growth but significantly inhibits overall *Chlorella vulgaris* cellular proliferation.

### 3.3. Influence of Dissolved Gases in Culturing Medium

**Figure 4:**
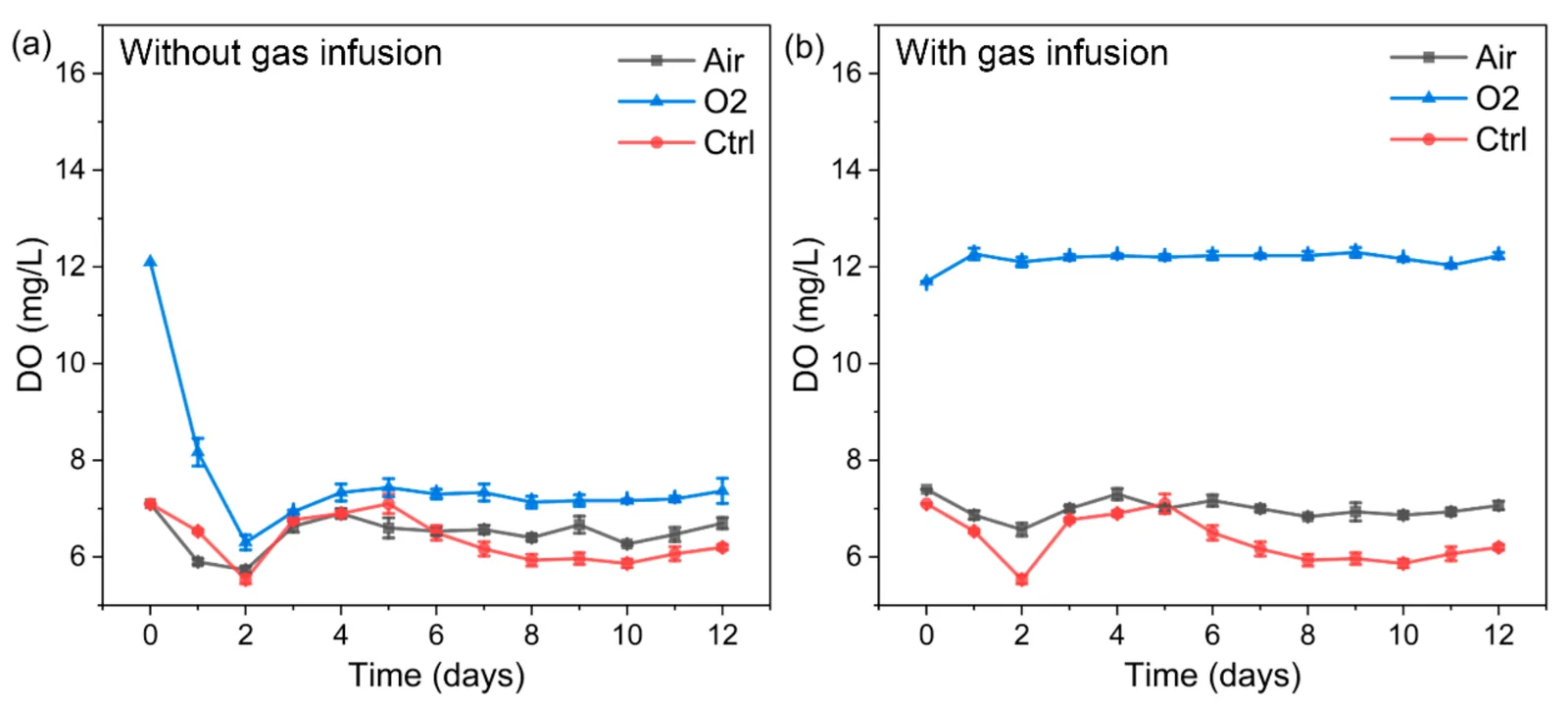
Daily monitored DO levels (**a**) without gas infusion, and (**b**) with daily gas infusion for air and oxygen nanobubble groups.

Figure 4 shows the temporal trends in DO concentrations in the culture media with and without daily gas infusion. Each data point represents the mean value of three replicates, with error bars indicating the standard error of the mean. In the oxygen nanobubble group with daily infusion of pure oxygen, the DO concentration remained consistently high throughout the cultivation period, reaching approximately 12 mg/L immediately following oxygen infusion. However, continuous monitoring was not performed and therefore the persistence of oxygen supersaturation between infusion events could not be verified. In contrast, in the oxygen nanobubble group without gas infusion, the initial DO concentration was also 12 mg/L but rapidly declined to around 8 mg/L within the first 24 h and further dropped to a minimum of 6.2 mg/L at 48 h. Thereafter, the DO gradually increased and stabilized at around 7.5 mg/L by Day 4. In comparison, the air nanobubble group and the control group exhibited minimal fluctuations in DO, maintaining relatively stable concentrations between 6 and 7 mg/L throughout the entire cultivation period.

These results reflect the diffusion of freshly generated oxygen nanobubbles. At the time of generation, the DO in the culture medium reaches a supersaturation level, which subsequently declines rapidly due to gas diffusion or uptake by microalgal metabolism. The sharp decline in DO after 24 h indicates a high diffusion rate, although the solution may still remain slightly supersaturated. By 48 h, the DO levels dropped to 6.2 mg/L, indicating a transition to an undersaturated state, which can further influence the rapidly expanding algal population.

In contrast, the daily oxygen infusion can effectively maintain the initial supersaturated conditions established by nanobubble formation. The comparison of DO levels between Figure 4a,b shows that daily air infusion maintained slightly higher DO concentrations than in the non-infusion group. These findings suggest that controlled gas infusion is a practical and effective method for sustaining gas supersaturation in the culture medium.

Meanwhile, both the air nanobubble group and the control group operated under moderate DO conditions due to the absence of high-concentration oxygen input, which can facilitate a dynamic balance between photosynthetic oxygen generation and respiratory consumption. Such an equilibrium provided a more stable physiological environment, supporting continuous algal growth. Notably, in the control group, DO concentration reached a minimum (5.3 mg/L) on Day 2, suggesting an oxygen consumption peak during this time. This decline can be attributed to the lag phase in microalgal growth, during which cells adapt to the new environment while consuming oxygen through respiration [37]. After this lag phase, the microalgae resumed active photosynthesis and oxygen production, allowing the culture to enter a stable growth period.

**Figure 5:**
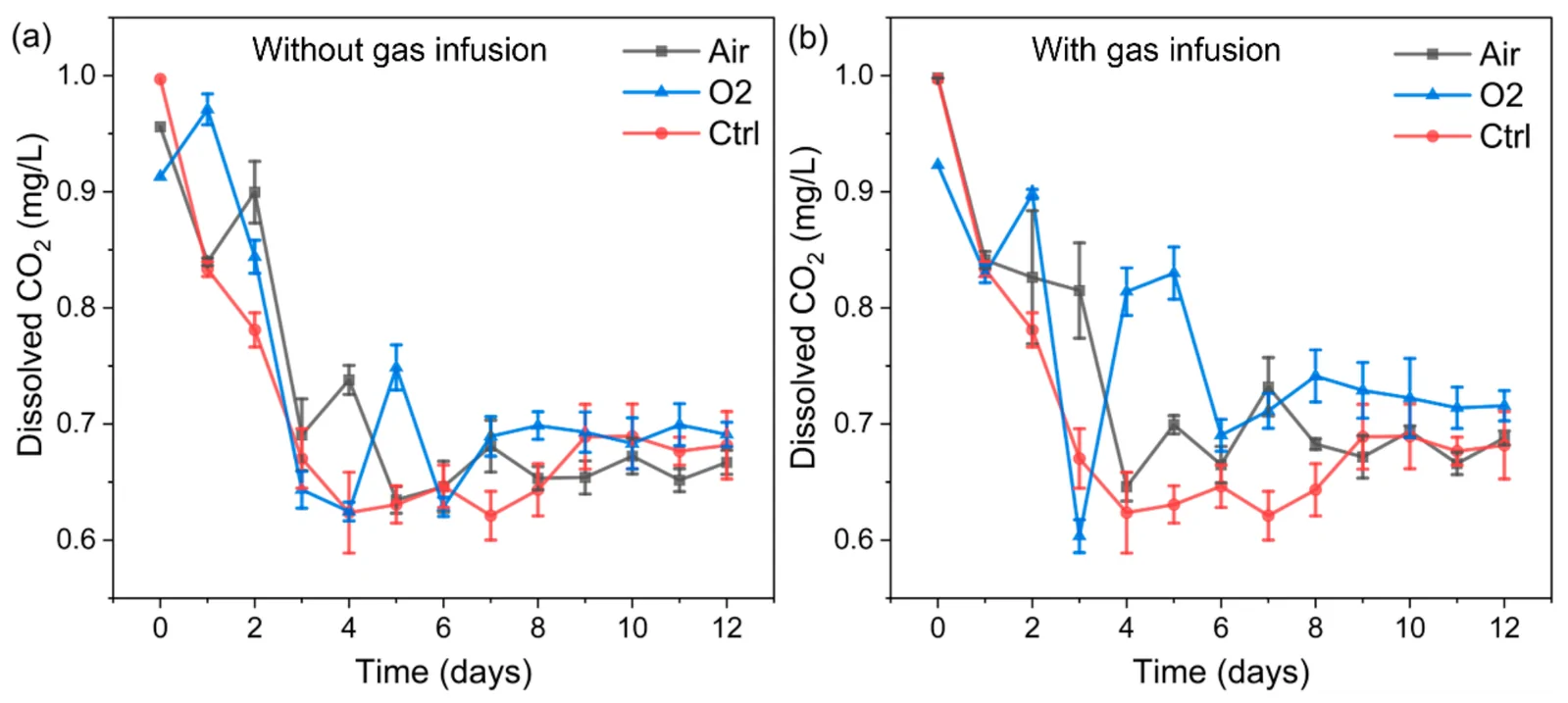
The dissolved CO_2_ in *Chlorella vulgaris* culture solutions (**a**) without gas infusion, and (**b**) with the daily gas infusion for the air and oxygen nanobubble groups.

We also monitored the daily change in dissolved CO_2_ across different experimental groups. Figure 5a,b exhibited similar trends in dissolved CO_2_ concentration over time for all experiment groups. The dissolved CO_2_ concentrations in the air nanobubble groups in the treatments without gas infusion are not significant from the control group with a *p*-value of 0.32 (ANOVA two-factor with replication *n* = 3, at 0.05 level). However, there are significant differences between the control and nanobubble groups in the treatment with gas infusion at the 0.01 level (*p* < 0.01, ANOVA two-factor with replication, *n* = 3). On Day 0, the freshly prepared *Chlorella vulgaris* cultures displayed an initial dissolved CO_2_ concentration of approximately 1 mg/L. This concentration gradually declined over the subsequent days, reaching a stable level of around 0.7 mg/L by Day 6. This trend is consistent with progressive consumption of dissolved CO2 during microalgal growth and the establishment of a dynamic balance between carbon uptake and release processes within the cultures. Notably, daily gas infusion with air did not lead to an increase in dissolved CO_2_ concentration in the culture media relative to the other treatments. Although significant differences in dissolved CO_2_ were observed among some treatments, dissolved CO_2_ represents only the CO_2_ in the culture medium available to microalgae.

## 4. Discussion

### 4.1. Oxygen-Mediated Inhibition of Microalgal Growth

In the present study, the growth delay of *Chlorella vulgaris* was observed in the oxygen nanobubble experiments with gas infusion coincided with elevated DO levels immediately post-infusion (~12 mg/L), whereas DO decreased rapidly in the absence of gas infusion. These observations suggest that oxygen supersaturation is the main driver of reduced cell density. In this study, the dissolved CO_2_ concentrations were maintained at similar levels across all experimental groups throughout the cultivation period. However, because bicarbonate, carbonate species, pH, and total inorganic carbon were not measured, these dissolved CO_2_ data alone are insufficient to fully evaluate carbon availability or limitation.

**Figure 6:**
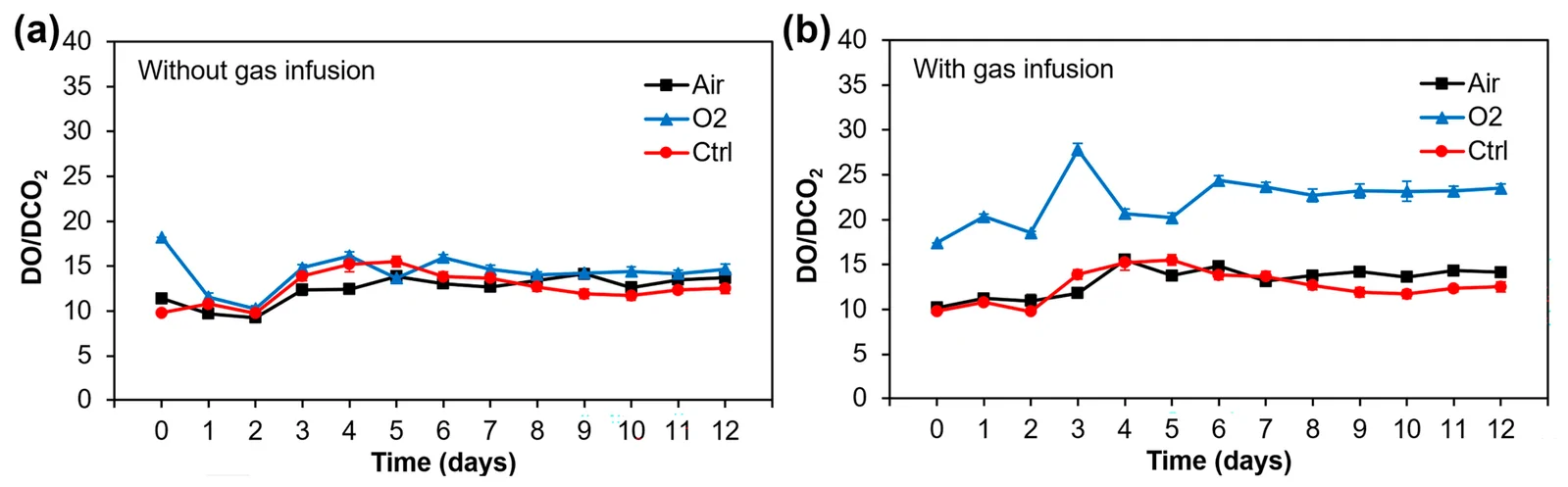
Dissolved O_2_/CO_2_ molar ratios (**a**) without gas infusion or (**b**) with gas infusion.

Microalgae generally grow well with DO concentrations close to air saturation, which is ~8 mg/L (0.2247 O2 mol/m^3^) [38]. Previous studies have reported growth inhibition when the DO exceeded air-saturation levels and the photosynthesis rate was reduced by 35% under pure oxygen, i.e., DO ~ 44 mg/L [39]. The observed growth of microalgae under high DO levels is consistent with previous studies reporting growth inhibition at the elevated DO levels in the culture media [20,21,40]. Borowitzka reported a huge decrease in photosynthesis at high DO levels in their study, and the DO, 150% of air saturation at equilibrium, corresponds to the dataset at a similar temperature [41]. Elevated oxygen concentrations can alter the balance between photosynthesis and photorespiration by increasing the O_2_/CO_2_ ratio surrounding microalgal cells [19]. Competing with carbon dioxide, oxygen preferred to bind with the Rubisco enzyme under high oxygen concentrations. This binding can negatively affect microalgal CO_2_ fixation to generate biomass by inhibition of photosynthesis [42]. In this study, high O_2_/CO_2_ mole ratios were found higher in the oxygen nanobubble group than the control group and air nanobubble group, maintaining stable levels even with oxygen daily infusion (Figure 6b). These high ratios may have favored photorespiration and contributed to the observed reduction in cell density accumulation.

The inhibitory effects are consistent with previous reports showing that oxygen supersaturation can reduce microalgal productivity through a combination of reduced carbon fixation efficiency and the associated enhancement of photorespiratory losses [19,20,21,43]. Therefore, a higher DO concentration is considered as a favorable factor that contributes to the observed reduced growth in the oxygen-enriched environment. Oxygen nanobubbles may establish a localized oxygen supersaturation near the gas–liquid interface. Previous studies have reported that reactive oxygen species (ROS) can be generated during hydrodynamic cavitation and bubble collapse [44,45] and hyperoxia conditions may induce oxidative stress in biological systems [46,47,48]. However, ROS production and associated oxidative damage were not measured in this study. Therefore, ROS-associated mechanisms will be considered in further investigation.

### 4.2. Role of Nanobubbles

While oxygen-driven inhibition provides the most direct explanation for the observed results, nanobubbles may still play an important role in modifying the gas environment. According to the diffusion theory, nanobubbles can facilitate rapid gas dissolution and promote the establishment of supersaturated conditions. In this study, initial oxygen nanobubble generation produced high DO levels (12 mg/L) that exceeded the normal DO saturation level (8 mg/L). However, without continuous gas infusion, these elevated DO levels dissipated within 24–48 h. In contrast, daily gas infusion treatment reduced algal growth.

These findings suggest that the main role of oxygen nanobubbles in this system may be to deliver rapid establishment of hyperoxia conditions, which can inhibit anaerobic organisms. Recent trials to control algal blooms using ozone nanobubbles have shown limited treatment success [3]. However, the failure outcomes can be attributed to several unknown factors, including nanobubble generation approach, generated ozone nanobubble concentrations, and the effective exposure of algal cells to ozone nanobubbles. In practical large-scale water treatment applications, e.g., lake cleaning, oxygen or ozone can be continuously introduced through nozzles or diffusers to generate nanobubbles in situ. Such an approach can quickly deliver oxygen supersaturation in aquatic systems, potentially enabling control of algal growth. However, the biological response might be delayed due to some uncontrolled complex environment conditions such as the nutrient factors.

### 4.3. Study Limitations and Implications for Future Research

Several limitations of this study should be noted. First, nanobubble stability and persistence were only characterized at the initial stage, and their presence during prolonged cultivation was not verified. However, the DO measurement from the groups without gas infusion suggest that nanobubbles can be effective in the first 2 days. Second, the use of a nanoporous gas diffuser in the daily gas infusion experiments precludes certainty in maintaining the concentration and size of the nanobubbles remaining in the system. Third, the experimental design did not infuse gas in the control as a nanobubble-free control. Because previous studies reported nanobubble generation through nanoporous diffusers [27,49,50], infusion of gas will generate nanobubbles. Therefore, the control infused gas may not be suitable as a nanobubble-free control. Achieving an identical DO profile using a different oxygenation technology (e.g., coarse-bubble aeration, headspace oxygenation, or membrane gas exchange) would nucleate new nanobubbles in oversaturated water and that would alter other mass-transfer characteristics, including bubble size, oxygen-transfer effectiveness, mixing conditions, and local microenvironments, e.g., the gas component in headspace oxygenation. Such a control would introduce additional experimental variables and would not isolate a single factor.

Despite these limitations, the study demonstrates that post-infusion oxygen supersaturation can inhibit microalgal growth and highlights the potential use of nanobubbles for controlling algal proliferation. Future studies should focus on in situ observations and evaluate system performance under environmentally relevant conditions, such as a pond, by direct generating oxygen or ozone nanobubbles for real applications to control algae and cyanobacteria bloom.

## 5. Conclusions

This study investigated the effects of the enrichment of dissolved oxygen mediated by air and oxygen nanobubble systems on the growth of the algae *Chlorella vulgaris*. The initial results indicated that in the absence of daily gas infusion, both the air and oxygen nanobubble groups caused a temporary delay in the algal growth. A limitation of the present study is that the experimental design could not separate nanobubble-specific effects from the effects of elevated dissolved oxygen concentrations. Therefore, the observed growth responses may be attributable to elevated dissolved oxygen, nanobubbles, or a combination of both factors. With daily gas infusion, the oxygen-infused treatment showed a lower cell density than the air-infused group. Dissolved gas analysis confirmed elevated dissolved oxygen levels and high O_2_/CO_2_ ratios in the oxygen nanobubble group. These conditions are consistent with previous reports about reductions in microalgal productivity under hyperoxia environments. Overall, these findings demonstrate that oxygen-enriched conditions generated using the nanobubble generation approach were associated with reduced microalgal growth. This preliminary study provides a basis for oxygen nanobubble technology as a potential tool for algal bloom in aquatic ecosystems. Future studies shall investigate the underlying mechanisms and examine appropriate engineering controls for large-scale systems relevant to water management applications.

## Data Availability

Dataset available on request from the authors.

## Supplementary Materials

The following supporting information can be downloaded at: https://www.mdpi.com/article/10.3390/mi17101169/s1, Table S1. Anova: Two-Factor with Replication, Air vs Ctrl (no gas infusion). Table S2. Anova: Two-Factor with Replication, O2 vs Ctrl (no gas infusion). Table S3. Anova: Two-Factor with Replication, Air vs O2 (no gas infusion). Table S4. Anova: Two-Factor with Replication, Air vs Ctrl (gas infusion). Table S5. Anova: Two-Factor with Replication, O2 vs Ctrl (gas infusion). Table S6. Anova: Two-Factor with Replication, Air vs O2 (gas infusion). Table S7. Interaction p-value for repeated measures. Table S8. Effective size (partial η^2^). Table S9. Cell density (cells/mL, no gas infusion) and standard error of the mean (SEM). Table S10. Cell density (cells/mL, gas infusion) and standard error of the mean (SEM).

## Abbreviations

The following abbreviations are used in this manuscript:

DO	Dissolved oxygen
ROS	Reactive oxygen species
