# Supplementary material for: The Effects of Nanobubble-Assisted Oxygen Supersaturation on the Growth of Chlorella vulgaris
Source: Micromachines (Basel). 2026 Oct 7;17(10):1169. doi: 10.3390/mi17101169 (PMC13707445; doi:10.3390/mi17101169)
Supplement: Supplementary file 1 [file micromachines-17-01169-s001.zip › micromachines-4458622-supplementary.pdf]

The Effects of nanobubble assisted oxygen supersaturation on the growth of *Chlorella vulgaris*

Lingxi Ouyang [1], Du Tuan Tran [1], Haotian Cha [2], Qin Li [2], Beng Hau Tan [3], Nam-Trung Nguyen [2,*] and Hongjie An [1,*]

[1] School of Environment and Science, Griffith University, 170 Kessels Road, Nathan, QLD 4111, Australia; oylx123321@gmail.com (L.O.); dutuan.tran@griffith.edu.au (D.T.T.)
[2] School of Engineering and Built Environment, Griffith University, 170 Kessels Road, Nathan, QLD 4111, Australia; h.cha@griffith.edu.au (H.C.); qin.li@griffith.edu.au (Q.L.)
[3] Shenzhen Institute for Advanced Study, University of Electronic Science and Technology of China, Shenzhen 518110, China; benghau@uestc.edu.cn (B.H.T.)
* Correspondence: nam-trung.nguyen@griffith.edu.au (N.-T.N.); hongjie.an@griffith.edu.au (H.A.)

Table S1. Anova: Two-Factor with Replication, Air vs Ctrl (no gas infusion)
Table S2. Anova: Two-Factor with Replication, O_2 vs Ctrl (no gas infusion)
Table S3. Anova: Two-Factor with Replication, Air vs O_2 (no gas infusion)
Table S4. Anova: Two-Factor with Replication, Air vs Ctrl (gas infusion)
Table S5. Anova: Two-Factor with Replication, O_2 vs Ctrl (gas infusion)
Table S6. Anova: Two-Factor with Replication, Air vs O_2 (gas infusion)
Table S7. Interaction p-value for repeated measures
Table S8. Effective size (partial η^2)
Table S9. Cell density (cells/mL, no gas infusion) and standard error of the mean (SEM)
Table S10. Cell density (cells/mL, gas infusion) and standard error of the mean (SEM)

Table S1. Anova: Two-Factor with Replication, Air vs Ctrl (no gas infusion)

Source of Variation	*SS*	*df*	*MS*	*F*	*P-value*	*F crit*
Sample (Treatment)	1.64×10^{13}	1	1.64×10^{13}	48.06	6.34×10^{-9}	4.03
Columns (Time)	3.59×10^{14}	12	2.99×10^{13}	87.59	4.34×10^{-30}	1.94
Interaction	1.1×10^{13}	12	9.13×10^{11}	2.67	0.0071	1.94
Within	1.78×10^{13}	52	3.42×10^{11}			
Total	4.04×10^{14}	77				

Table S2. Anova: Two-Factor with Replication, O_2 vs Ctrl (no gas infusion)

Source of Variation	*SS*	*df*	*MS*	*F*	*P-value*	*F crit*
Sample (Treatment)	1.77×10^{13}	1	1.77×10^{13}	51.97	2.30×10^{-9}	4.03
Columns (Time)	3.68×10^{14}	12	3.07×10^{13}	90.08	2.18×10^{-30}	1.94
Interaction	8.62×10^{12}	12	7.18×10^{11}	2.11	0.032	1.94
Within	1.77×10^{13}	52	3.41×10^{11}			
Total	4.12×10^{14}	77				

Table S3. Anova: Two-Factor with Replication, Air vs O_2 (no gas infusion)

Source of Variation	*SS*	*df*	*MS*	*F*	*P-value*	*F crit*
Sample (Treatment)	2.38×10^{10}	1	2.38×10^{10}	1.42	0.24	4.03
Columns (Time)	3.73×10^{14}	12	3.11×10^{13}	1856.30	5.91×10^{-64}	1.94
Interaction	7.43×10^{11}	12	6.19×10^{10}	3.70	0.00047	1.94
Within	8.7×10^{11}	52	1.67×10^{10}			
Total	3.74×10^{14}	77				

Table S4. Anova: Two-Factor with Replication, Air vs Ctrl (gas infusion)

Source of Variation	*SS*	*df*	*MS*	*F*	*P-value*	*F crit*
Sample (Treatment)	1.15×10^{13}	1	1.15×10^{13}	32.00	6.66×10^{-7}	4.03
Columns (Time)	3.72×10^{14}	12	3.1×10^{13}	86.10	6.61×10^{-30}	1.94
Interaction	9.59×10^{12}	12	7.99×10^{11}	2.22	0.024	1.94
Within	1.87×10^{13}	52	3.6×10^{11}			
Total	4.12×10^{14}	77				

Table S5. Anova: Two-Factor with Replication, O_2 vs Ctrl (gas infusion)

Source of Variation	*SS*	*df*	*MS*	*F*	*P-value*	*F crit*
Sample (Treatment)	6.13×10^{13}	1	6.13×10^{13}	162.45	1.26×10^{-17}	4.02
Columns (Time)	2.45×10^{14}	12	2.04×10^{13}	54.00	5.17×10^{-25}	1.94
Interaction	1.55×10^{13}	12	1.29×10^{12}	3.41	0.0010	1.94
Within	1.96×10^{13}	52	3.78×10^{11}			
Total	3.41×10^{14}	77				

Table S6. Anova: Two-Factor with Replication, Air vs O_2 (gas infusion)

Source of Variation	*SS*	*df*	*MS*	*F*	*P-value*	*F crit*
Sample (Treatment)	1.97×10^{13}	1	1.97×10^{13}	274.03	2.24×10^{-22}	4.03
Columns (Time)	2.52×10^{14}	12	2.1×10^{13}	291.68	3.18×10^{-43}	1.94
Interaction	1.65×10^{13}	12	1.37×10^{12}	19.10	5.58×10^{-15}	1.94
Within	3.74×10^{12}	52	7.19×10^{10}			
Total	2.91×10^{14}	77				

Table S7. Interaction p-value for repeated measures using mixed model: Cell density ~ Treatment × Day + (1|Flask)

Day	gas infusion			no gas infusion		
	Air vs Ctrl	O_2 vs Ctrl	Air vs O_2	Air vs Ctrl	O_2 vs Ctrl	Air vs O_2
1	0.893	0.66	0.648	0.864	0.752	0.689
2	0.0253	0.0139	0.668	0.0124	0.00267	0.157
3	0.000524	0.00117	0.859	3.73×10^{-6}	9.11×10^{-8}	0.0424
4	4.83×10^{-5}	5.90×10^{-6}	0.386	1.42×10^{-9}	2.49×10^{-7}	0.0177
5	1.62×10^{-7}	1.56×10^{-10}	0.0517	5.21×10^{-15}	4.56×10^{-11}	0.00101
6	1.93×10^{-5}	7.82×10^{-11}	4.89×10^{-4}	1.11×10^{-9}	5.06×10^{-7}	0.00437
7	5.91×10^{-6}	4.76×10^{-18}	2.86×10^{-10}	7.98×10^{-5}	7.27×10^{-5}	0.909
8	0.0139	1.83×10^{-10}	3.64×10^{-9}	0.0217	0.182	0.00929
9	0.193	1.38×10^{-8}	6.61×10^{-11}	0.0476	0.154	0.135
10	0.573	5.91×10^{-11}	9.49×10^{-26}	0.110	0.0582	0.402
11	0.611	9.66×10^{-9}	3.09×10^{-20}	0.327	0.389	0.753
12	0.915	5.32×10^{-11}	1.25×10^{-21}	0.0906	0.0619	0.617

Table S8. Effective size (partial $\eta^2 = SS_{treatment}/(SS_{treatment} + SS_{within})$)

Comparison	$SS_{treatment}$	SS_{within}	p-value	partial η^2
Air vs Ctrl (gas infusion)	1.15×10^{13}	1.87×10^{13}	6.66×10^{-7}	0.38
O_2 vs Ctrl (gas infusion)	6.13×10^{13}	1.96×10^{13}	1.26×10^{-17}	0.76
Air vs O_2 (gas infusion)	1.97×10^{13}	3.74×10^{12}	2.24×10^{-22}	0.84
Air vs Ctrl (no gas infusion)	1.64×10^{13}	1.78×10^{13}	6.34×10^{-9}	0.48
O_2 vs Ctrl (no gas infusion)	1.77×10^{13}	1.77×10^{13}	2.30×10^{-9}	0.5
Air vs O_2 (no gas infusion)	2.38×10^{10}	8.7×10^{11}	0.24	0.027

Table S9. Cell density (cells/mL, no gas infusion) and standard error of the mean (SEM)

Day	Ctrl_Mean	SEM	Air_Mean	SEM	O_2_Mean	SEM
0	1255385	99017.15	910000	63034.38	1313333	84721.77
1	1226000	95937.33	976666.7	38355.07	1226667	115720
2	2405000	227702.9	1491667	63530.4	1665000	70415.43
3	3333333	338695.2	1536667	89950.6	1916667	85035.94
4	3952500	381205.8	2590000	101127	2416667	129348.8
5	4790769	395541.5	3323333	164370.9	3043333	107662
6	6006667	434687	4266667	202758.8	4016667	113773.7
7	7183333	621086	5866667	149814.7	5983333	286841
8	6958333	605203.6	6483333	157938.1	6283333	312427.8
9	6869231	583011.8	6250000	189296.9	6216667	260021.4
10	7078571	583748.2	6300000	220605.2	6500000	173205.1
11	6800000	556495.5	6456667	143728.1	6523333	154783
12	7041667	650854.1	6396667	147595.5	6558333	119147.4

Table S10. Cell density (cells/mL, gas infusion) and standard error of the mean (SEM)

Day	Air_Mean	SEM	Ctrl_Mean	SEM	O_2_Mean	SEM
0	1203333	83768.03	1255385	99017.15	1221278	85924.39
1	1124278	77471.14	1226000	95937.33	1031833	84780.31
2	1573833	121074.4	2405000	227702.9	1488167	121104.4
3	2075000	152204.9	3333333	338695.2	2136000	166030.7
4	2822500	243375	3952500	381205.8	2630667	192741.9
5	3604167	293726.5	4790769	395541.5	3151667	293762.4
6	4383333	396094.7	6006667	434687	3558333	314873.5
7	5556667	456696.2	7183333	621086	4050000	354835.3
8	6050000	499991.5	6958333	605203.6	4641667	404290.6
9	6285556	426419.6	6869231	583011.8	4725000	403722
10	7093333	569646.4	7078571	583748.2	4575000	473338.3
11	6901667	532589.6	6800000	556495.5	4691667	411701.2
12	6950000	582838.6	7041667	650854.1	4658333	420647.6
